# Retraction: Inhibition of reactive gliosis prevents neovascular growth in the mouse model of oxygen-induced retinopathy

**DOI:** 10.1371/journal.pone.0227156

**Published:** 2019-12-19

**Authors:** The PLOS ONE Editors

After this article [1] was published, concerns were raised about results reported in Figs 5 and 7.

Specifically:

- In the left panels in Fig 5, similarities were noted between the elongated GFAP-stained cells sections in the non-treated Oxygen-injured retinas and in the DMSO-treated Oxygen-injured retinas.
- In Fig 5, there appears to be a region of overlap in the left panels shown for non-treated Oxygen-injured retina (see area marked as B) and the DMSO-treated Oxygen-injured retina (see area marked as A).
- Concerns were raised about the quality and presentation of western blot data reported in Fig 7. Given the brightness/contrast levels of images in this figure and the vertical lines separating lanes, one cannot confirm the integrity of the images or clarify whether the lanes for each experiment show data from the same blot and exposure. In addition, there appear to be horizontal discontinuities below bands shown in lanes 2, 3 of the upper GFAP panel, and vertical discontinuities around the bands shown in both β-actin panels.

The authors were unable to resolve these issues or provide the original data underlying the results in response to journal queries. Without the original data we cannot resolve the above concerns which call into question the validity of the reported results.

In light of these concerns, the *PLOS ONE* Editors retract this article.

Per FAAM and FHAM, the underlying data for this study were held by MD for whom current contact information is not available. The journal has not received confirmation that MD received communications about this case.

FHAM and FAAM replied to our notification but did not specify agreement or disagreement with the retraction. MD either could not be reached or did not reply.
